# Assessing the prior event rate ratio method via probabilistic bias analysis on a Bayesian network

**DOI:** 10.1002/sim.8435

**Published:** 2019-12-01

**Authors:** Edward W. Thommes, Salaheddin M. Mahmud, Yinong Young‐Xu, Julia Thornton Snider, Robertus van Aalst, Jason K.H. Lee, Yuliya Halchenko, Ellyn Russo, Ayman Chit

**Affiliations:** ^1^ Sanofi Pasteur Swiftwater Pennsylvania; ^2^ Department of Mathematics and Statistics University of Guelph Guelph Ontario Canada; ^3^ Department of Community Health Sciences, College of Medicine University of Manitoba Winnipeg Manitoba Canada; ^4^ Clinical Epidemiology Program Veterans Affairs Medical Center White River Junction Vermont; ^5^ Department of Psychiatry Geisel School of Medicine at Dartmouth Hanover New Hampshire; ^6^ Precision Health Economics Oakland California; ^7^ Leslie Dan School of Pharmacy University of Toronto Toronto Ontario Canada; ^8^ Sanofi Pasteur Toronto Ontario Canada

**Keywords:** Bayesian networks, observational studies, probabilistic bias analysis, prior event rate ratio (PERR), unmeasured confounders

## Abstract

**Background:** Unmeasured confounders are commonplace in observational studies conducted using real‐world data. Prior event rate ratio (PERR) adjustment is a technique shown to perform well in addressing such confounding. However, it has been demonstrated that, in some circumstances, the PERR method actually increases rather than decreases bias. In this work, we seek to better understand the robustness of PERR adjustment.

**Methods:** We begin with a Bayesian network representation of a generalized observational study, which is subject to unmeasured confounding. Previous work evaluating PERR performance used Monte Carlo simulation to calculate joint probabilities of interest within the study population. Here, we instead use a Bayesian networks framework.

**Results:** Using this streamlined analytic approach, we are able to conduct probabilistic bias analysis (PBA) using large numbers of combinations of parameters and thus obtain a comprehensive picture of PERR performance. We apply our methodology to a recent study that used the PERR in evaluating elderly‐specific high‐dose (HD) influenza vaccine in the US Veterans Affairs population. That study obtained an HD relative effectiveness of 25% (95% CI: 2%‐43%) against influenza‐ and pneumonia‐associated hospitalization, relative to standard‐dose influenza vaccine. In this instance, we find that the PERR‐adjusted result is more like to underestimate rather than to overestimate the relative effectiveness of the intervention.

**Conclusions:** Although the PERR is a powerful tool for mitigating the effects of unmeasured confounders, it is not infallible. Here, we develop some general guidance for when a PERR approach is appropriate and when PBA is a safer option.

## 1 INTRODUCTION

From healthcare to econometrics to the social sciences, the findings of observational studies almost always suffer from bias due to confounding. Numerous methods to control for confounding have been developed, and for each of these, dealing with unmeasured confounders usually presents the trickiest challenge.

One method that has been used to address confounding due to unmeasured variables is prior event rate ratio (PERR) adjustment, developed by Weiner and colleagues and shown to perform well in reproducing the results of a Scandinavian randomized controlled trial (RCT) with an observational study conducted using UK Electronic Medical Record (EMR) data.^1^ Subsequent work by Weiner et al showed a similarly good performance of PERR applied to observational studies in replicating other RCTs.^2^, ^3^, ^4^ Uddin et al^5^ studied the performance of PERR and identified situations in which PERR adjustment increases rather than decreases bias. Further development and refinement, including derivation from PERR adjustment of a pairwise Cox likelihood function, was carried out by Lin and Henley.^6^

**Figure 1:**
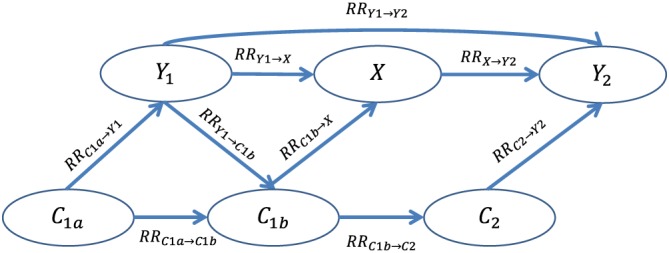
Bayesian network, or probabilistic directed acyclic graph, with dichotomous variables denoting baseline period event *Y*_1_, treatment *X*, observation‐period event *Y*_2_, time‐varying unobserved confounder {*C*_1*a*_, *C*_1*b*_, *C*_2_}, and the rate ratios (RRs) describing the associations among them. The RRs are defined in Equations (10) to (18) [Colour figure can be viewed at http://wileyonlinelibrary.com]

Here, we endeavor to understand when PERR yields meaningful bounds on the size and/or direction of the treatment effect of interest, even if it cannot be guaranteed to remove (or even reduce) bias. We begin with a Bayesian network^7^, ^8^ representation of the causal pathways in a standard observational study (Figure 1). We use Bayesian network analysis to define exact analytic expressions for the joint probabilities of study variables. (Uddin et al used Monte Carlo simulation to approximate the joint probabilities.) We examine the behavior of the system and identify circumstances where the PERR method overestimates and underestimates the true effect of a treatment. As an illustrative example, we then apply our approach to performing a probabilistic bias analysis (PBA) of a study of the relative effectiveness of high‐dose (HD) versus standard‐dose (SD) seasonal influenza vaccine in the Veterans Affairs (VA) patient population.^9^

## 2 THE PERR METHOD

Let us suppose we are conducting a cohort study in which the rate of the outcome of interest is measured not just during the observation period after the treatment but also during a baseline period before the treatment. We denote a possible event occurring during the baseline period as *Y*_1_, a possible event occurring during the observation period as *Y*_2_ and a possible treatment as *X*. The variables are dichotomous, so, for example, an individual with $\left(Y_{1}=y_{1},X=\bar{x},Y_{2}=\bar{y_{2}}\right)$ experienced an event in the baseline period, did not receive the treatment and did not experience an event in the observation period. To streamline the notation, we will write *Y*_1_ = *y*_1_ as *y*_1_, etc. We use *P* to denote the probability, over a time interval *T*, that an event occurs in an individual. For example, *P*(*y*_2_| *x*) is the probability per time *T* that an individual experiences an event in the observation period, conditional on having received treatment. Measured at the population level, *P* becomes the average incidence rate, ie, events per person per time *T*. We denote the incidence rate as *R*. Since the two quantities are numerically identical (assuming that both are defined over the same unit of time, and that individuals are independent), we will use them interchangeably. The incidence rate ratio (RR) in the baseline period is then

(1) $$RR_{\text{base}}=\frac{R\left(y_{1}|x\right)}{R\left(y_{1}|\bar{x}\right)},$$

whereas, in the observation period, it is

(2) $$RR_{\text{obs}}=\frac{R\left(y_{2}|x\right)}{R\left(y_{2}|\bar{x}\right)}.$$

Now, let us suppose that we find *RR*_*base*_ for the event to differ from unity by a statistically significant amount. This suggests that the treatment and control arms are unbalanced with respect to the distribution of one or more determinants of the event. Assuming that there are no systematic measurement errors and that all measurable confounders have already been controlled for, we are led to suspect that there is residual unmeasured confounding by indication. The PERR method attempts to correct for the imbalance and thus recover the “true” RR of the treatment through normalizing *RR*_*obs*_ by *RR*_*base*_

(3) $$\text{PERR}=\frac{RR_{\text{obs}}}{RR_{\text{base}}}.$$

## 3 THE CAUSAL MODEL

We begin with a Bayesian network (or probabilistic directed acyclic graph [DAG])^7^ depicting, from the perspective of a single individual, the potential causal associations in our study. We include an unmeasured dichotomous confounder, $C\in \left\{c,\bar{c}\right\}$. We define distinct values for the confounder in the baseline period (*C*_1_) and the observation period (*C*_2_). We further divide *C*_1_ into *C*_1*a*_ and *C*_1*b*_ to allow for the possibility that the relationship between *C*_1_ and *Y*_1_ is bidirectional (ie, the state of *C*_1_ influences the state of *Y*_1_, and the state of *Y*_1_ influences the state of *C*_1_). We also allow for the possibility of a direct causal connection between baseline event *Y*_1_ and treatment *X*. One can show (see the work of Greenland et al^7^) that this DAG has a null adjustment set and that there exist no instruments or conditional instruments that might permit instrumental variable regression.

We write down a set of equations that describe this causal diagram. The effect of each directed edge is expressed as an RR whose value depends on the state of the variable corresponding to the edge's originating vertex. The model equations describing the population‐level incidence rates *R* (or equivalently, individual‐level probabilities *P*) over a time period *T* of the occurrence of *c*, *y*_1_, *x*, and *y*_2_ are

(4) $$\;R\left(C_{1a}=c_{1a}\right)=P\left(C_{1a}=c_{1a}\right)=r_{c1a}$$

(5) $$\;R\left(Y_{1}=y_{1}|C_{1a}\right)=P\left(Y_{1}=y_{1}|C_{1a}\right)=\Pi_{\left[0,1\right]}\left(r_{y1}\cdot RR_{C1a\to Y1}\right)$$

(6) $$\;R\left(C_{1b}=c_{1b}|C_{1a},Y_{1}\right)=P\left(C_{1b}=c_{1b}|C_{1a},Y_{1}\right)=\Pi_{\left[0,1\right]}\left(r_{c1b}RR_{Y1\to C1b}RR_{C1a\to C1b}\right)$$

(7) $$\;R\left(X=x|C_{1b},Y_{1}\right)=P\left(X=x|C_{1b},Y_{1}\right)=\Pi_{\left[0,1\right]}\left(r_{x}\cdot RR_{C1b\to X}\cdot RR_{Y1\to X}\right)$$

(8) $$\;R\left(C_{2}=c_{2}|C_{1b}\right)=P\left(C_{2}=c_{2}|C_{1b}\right)=\Pi_{\left[0,1\right]}\left(r_{c2}\cdot RR_{C1b\to C2}\right)$$

(9) $$R\left(Y_{2}=y_{2}|C_{2},Y_{1},X\right)=P\left(Y_{2}=y_{2}|C_{2},Y_{1},X\right)=\Pi_{\left[0,1\right]}\left(r_{y2}\cdot RR_{C2\to Y2}\cdot RR_{Y1\to Y2}\cdot {\mathrm{RR}}_{X\to Y2}\right),$$

where *r*_*c*1*a*_, *r*_*y*1_, *r*_*c*1*b*_, *r*_*x*_, *r*_*c*2_, *r*_*Y*2_ are constants on [0,1]. The operator Π_[0,1]_(*x*), ∈ *ℝ*, is the closest‐element mapping from real numbers to [0,1], ie,

Π0,1x=0,ifx<0x,ifx∈0,11,ifx>1.

The *RR*s are defined as follows:

(10) RRC1a→Y1=Fc1a→Y1,ifC1a=c1a1,ifC1a=c1a‾

(11) RRY1→C1b=FY1→C1b,ifY1=y11,ifY1=y1‾

(12) RRC1a→C1b=Fc1a→C1b,ifC1a=c1a1,ifC1a=c1a‾

(13) RRC1b→X=Fc1b→X,ifC1b=c1b1,ifC1b=c1b‾

(14) RRY1→X=Fy1→X,ifY1=y11,ifY1=y1‾

(15) RRC1b→C2=Fc1b→C2,ifC1b=c1b1,ifC1b=c1b‾

(16) RRC2→Y2=Fc2→Y2,ifC2=c21,ifC2=c2‾

(17) RRY1→Y2=Fy1→Y2,ifY1=y11,ifY1=y1‾

(18) RRX→Y2=Fx→Y2,ifX=x1,ifX=x‾,

where the *F*'s are constants >0.

Uddin et al used Monte Carlo simulation to obtain approximate conditional rates/probabilities from this model. Instead, we will use Bayesian network analysis (see, eg, the work of Pearl^8^) to calculate exact probabilities, as follows.

The incidence rate with which a given set of values of *C*_1*a*_, *Y*_1_, *C*_1*b*_, *X*, *C*_2_, and *Y*_2_ is realized on the above network is their joint probability

(19) $$\begin{array}{cc} & \;R\left(C_{1a},Y_{1},C_{1b},X,C_{2},Y_{2}\right)=P\left(C_{1a},Y_{1},C_{1b},X,C_{2},Y_{2}\right)\\ & =P\left(C_{1a}\right)P\left(Y_{1}|C_{1a}\right)P\left(C_{1b}|C_{1a}|Y_{1}\right)P\left(X|C_{1a},Y_{1},C_{1b}\right)P\left(C_{2}|C_{1a}|Y_{1}|C_{1b}|X\right)P\left(Y_{2}|C_{1a},Y_{1},C_{1b},X,C_{2}\right). \end{array}$$

Joint rates/probabilities of subsets of the variables are calculated by summing the joint probabilities over all possible combinations of the remaining variables, for example,

(20) $$R\left(y_{1},x\right)=P\left(y_{1},x\right)=\sum_{\left\{c_{1a},\bar{c_{1a}}\right\}}\sum_{\left\{c_{1b},\bar{c_{1b}}\right\}}\sum_{\left\{c_{2},\bar{c_{2}}\right\}}\sum_{\left\{y_{2},\bar{y_{2}}\right\}}P\left(C_{1a},Y_{1}=y_{1},C_{1b},X=x,C_{2},Y_{2}\right).$$

Conditional rates/probabilities can likewise be calculated, for example,

$$R\left(y_{1}|x\right)=P\left(y_{1}|x\right)=\frac{P\left(y_{1},x\right)}{P\left(x\right)},$$

where

$$R\left(x\right)=P\left(x\right)=\sum_{\left\{c_{1a},\bar{c_{1a}}\right\}}\sum_{\left\{y_{1},\bar{y_{1}}\right\}}\sum_{\left\{c_{1b},\bar{c_{1b}}\right\}}\sum_{\left\{c_{2},\bar{c_{2}}\right\}}\sum_{\left\{y_{2},\bar{y_{2}}\right\}}P\left(C_{1a},Y_{1},C_{1b},X=x,C_{2},Y_{2}\right).$$

We implement these calculations in the R language.^10^

## 4 THE PERFORMANCE OF PERR ADJUSTMENT

In an observational study, the following incidence rates will be measured:

- R(x), the rate of treatment across the study population;
- $R(y_{2}|x)$, the rate of the event in the treatment arm during the observation period;
- $R\left(y_{2}|\bar{x}\right)$, the rate of the event in the control arm during the observation period;
- $R(y_{2})$, the rate of the event across the whole study population during the observation period (where $R\left(y_{2}\right)=R\left(y_{2}|x\right)R\left(x\right)+R\left(y_{2}|\bar{x}\right)R\left(\bar{x}\right)$).

If patients have also been observed during a baseline period, then we further have measurements of the following:

- $R(y_{1}|x)$, the rate of the event in the treatment arm during the baseline period (ie, the rate among those who are later treated);
- $R\left(y_{1}|\bar{x}\right)$, the rate of the event in the control arm during the baseline period (ie, the rate among those who are not later treated);
- $R(y_{1})$, the rate of the event across the whole study population during the baseline period (where $R\left(y_{1}\right)=R\left(y_{1}|x\right)R\left(x\right)+R\left(y_{1}|\bar{x}\right)R\left(\bar{x}\right)$).

The RRs between treatment and control arms in the observation and baseline period are given by Equations (1) and (2), respectively, while the PERR estimate of the treatment effect is given by Equation (3).

The true, unbiased effect of treatment *x* on the probability of event *y*_2_ is given by *F*_*x* → *Y*2_ (see Equation (18)). The question we wish to answer for our causal model is, under what circumstances does PERR succeed in decreasing bias, and under what circumstances does it actually increase bias? Furthermore, under what circumstances is the *PERR* estimate either <*F*_*x* → *Y*2_ or >*F*_*x* → *Y*2_? For specificity, we take events *y* to be harmful, thus the smaller *F*_*x* → *Y*2_, the more effective the treatment *x*. Therefore, when *PERR*/*F*_*x* → *Y*2_ < 1, the effectiveness of *x* is overestimated (overoptimistic PERR), whereas if *PERR*/*F*_*x* → *Y*2_ > 1, it is underestimated (pessimistic PERR).

We begin by computing the behavior of the *PERR* in comparison to the true effect *F*_*x* → *Y*2_ under different scenarios.

**Figure 2:**
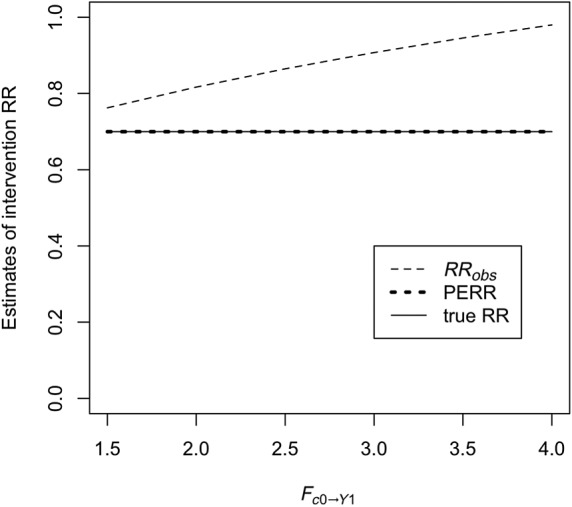
The conventional rate ratio (RR) (*RR*_*obs*_), the prior event rate ratio (PERR) estimator, and the true RR (=*F*_*x* − *Y*2_, set to 0.7) in the case where *C*_1*a*_ = *C*_1*b*_ = *C*_2_ and *F*_*c*2 → *Y*2_ = *F*_*c*1*a* → *Y*1_. *F*_*c*1*b* → *X*_ is held fixed at 1.5, and *r*_*c*1*a*_ = 0.25. Note that the PERR and the true RR are coincident

**Figure 3:**
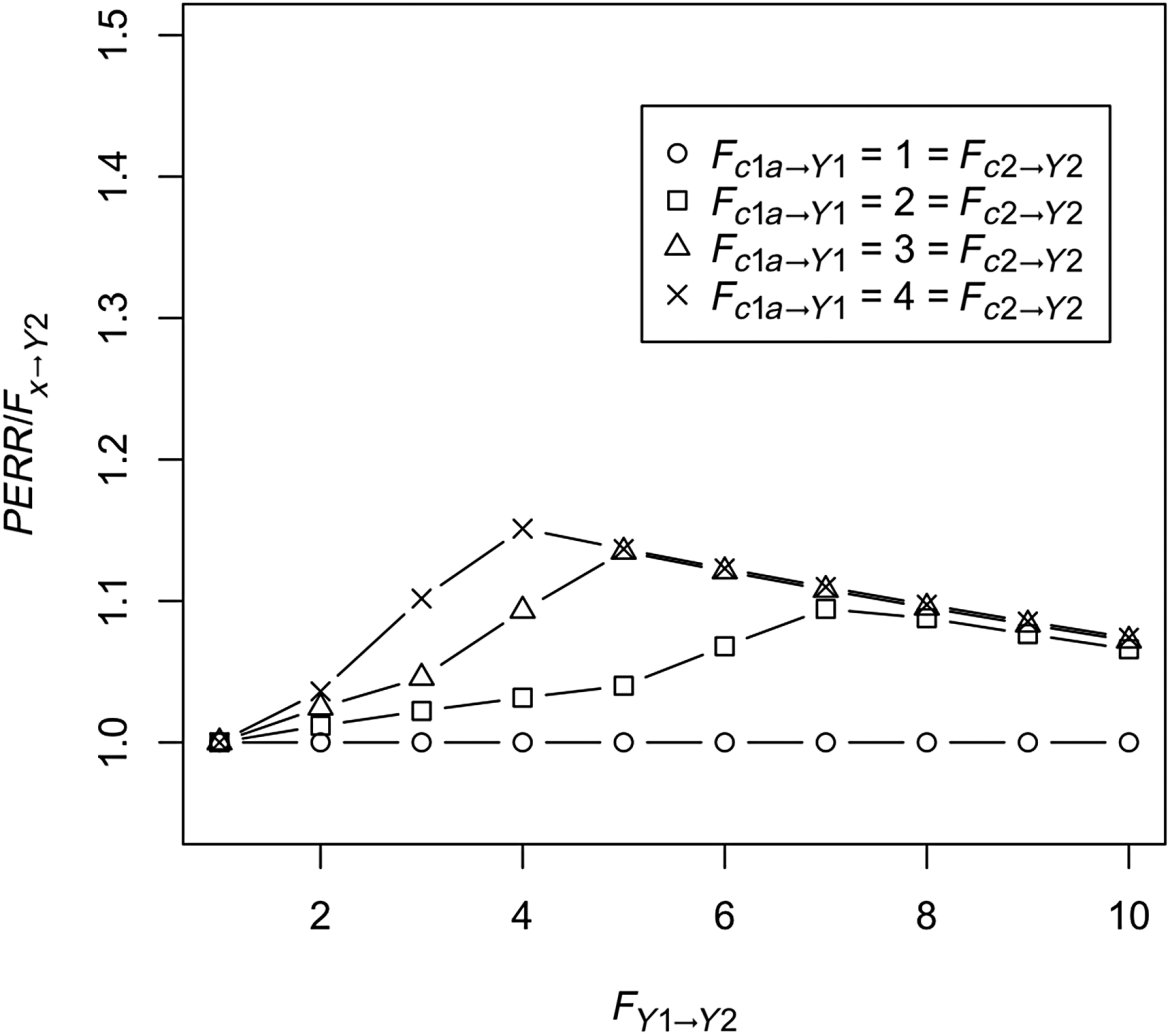
Performance of the prior event rate ratio (PERR) estimator as a function of *F*_*y*1 → *Y*2_. The ratio of the PERR to the true effect, ie, *F*_*x* → *Y*2_, is plotted. A ratio greater than 1 thus corresponds to an overestimate of the true RR and, hence, an underestimate of the true treatment effect. Scenarios with *F*_*c*1*a* → *Y*1_ = 1 (Scenario a), = 2 (Scenario b), = 3 (Scenario), and = 4 (Scenario d) are shown. As before, we set *C*_1*a*_ = *C*_1*b*_ = *C*_2_, *F*_*c*2 → *Y*2_ = *F*_*c*1*a* → *Y*1_, and fix *F*_*c*1*b* → *X*_ = 1.5, *r*_*c*1*a*_ = 0.25. In each case, the overestimate of the true rate ratio (RR) by the PERR initially becomes greater with *F*_*y*1 → *Y*2_, then reaches a maximum, and decreases again. The height of the maximum increases with *F*_*c*1*a* → *Y*1_. When *F*_*c*1*a* → *Y*1_ = 4, the maximum PERR overestimate of the true RR is 15% and occurs when *F*_*y*1 → *Y*2_ = 4

**Figure 4:**
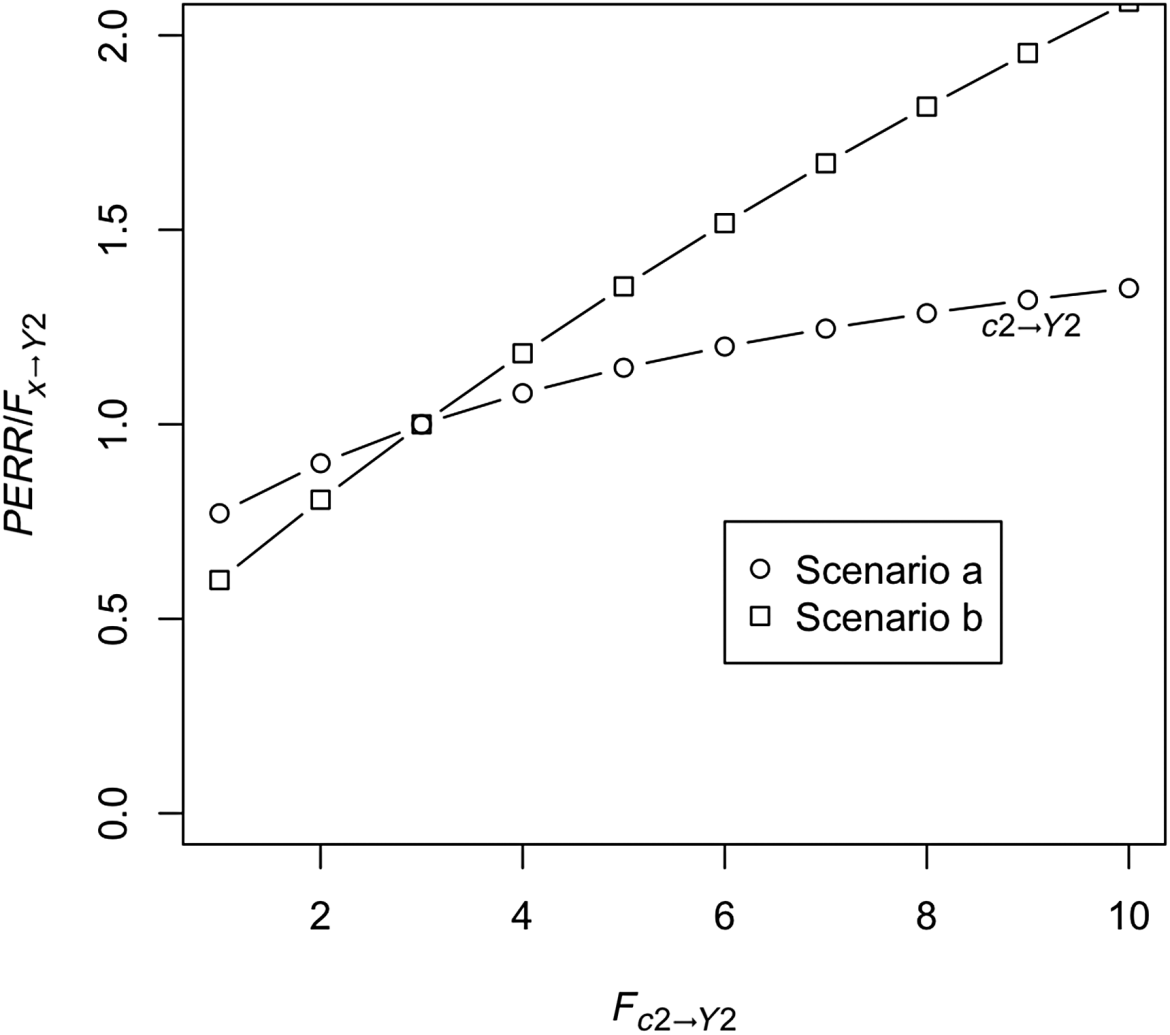
Accuracy of the prior event rate ratio (PERR) estimator as a function of *F*_*c*2 → *Y*2_, with *F*_*c*1*a* → *Y*1_ held fixed at 3. In Scenario a, *F*_*c*1*b* → *X*_ = 1.5, whereas in Scenario b, *F*_*c*1*b* → *X*_ = 1.9. As before, *r*_*c*1*a*_ = 0.25

**Figure 5:**
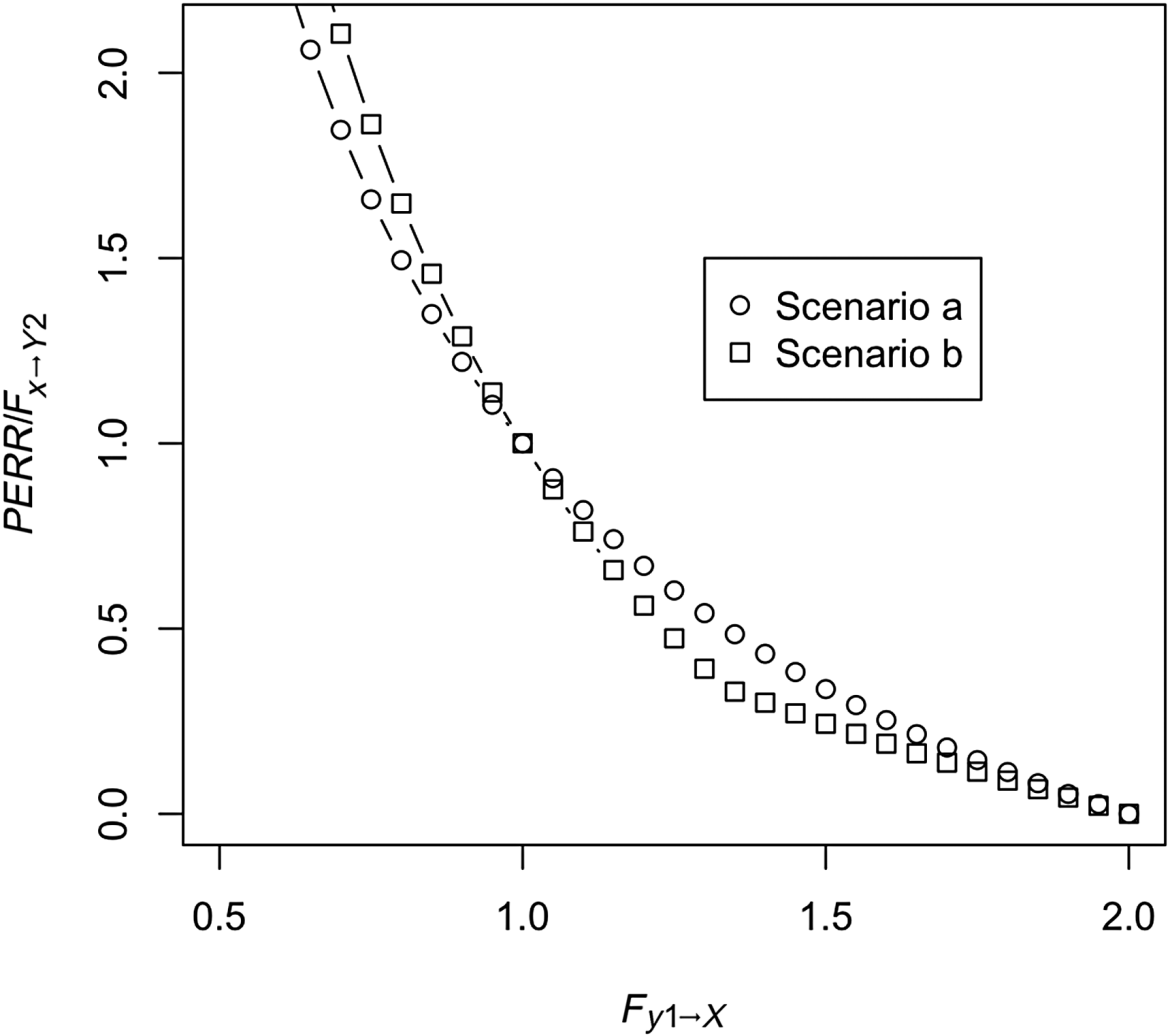
Performance of the prior event rate ratio (PERR) estimator when *F*_*y*1 → *X*_ differs from 1. In Scenario a, there are no confounders, ie, *r*_*c*1*a*_ = 0, and as before, *C*_2_ = *C*_1*b*_ = *C*_1*a*_. In Scenario b, the confounder is present (*r*_*c*1*a*_ = 0.25), with *F*_*c*2 → *Y*2_ = *F*_*c*1*a* → *Y*1_ = 3 and *F*_*c*1*b* → *X*_ = 1.5

**Figure 6:**
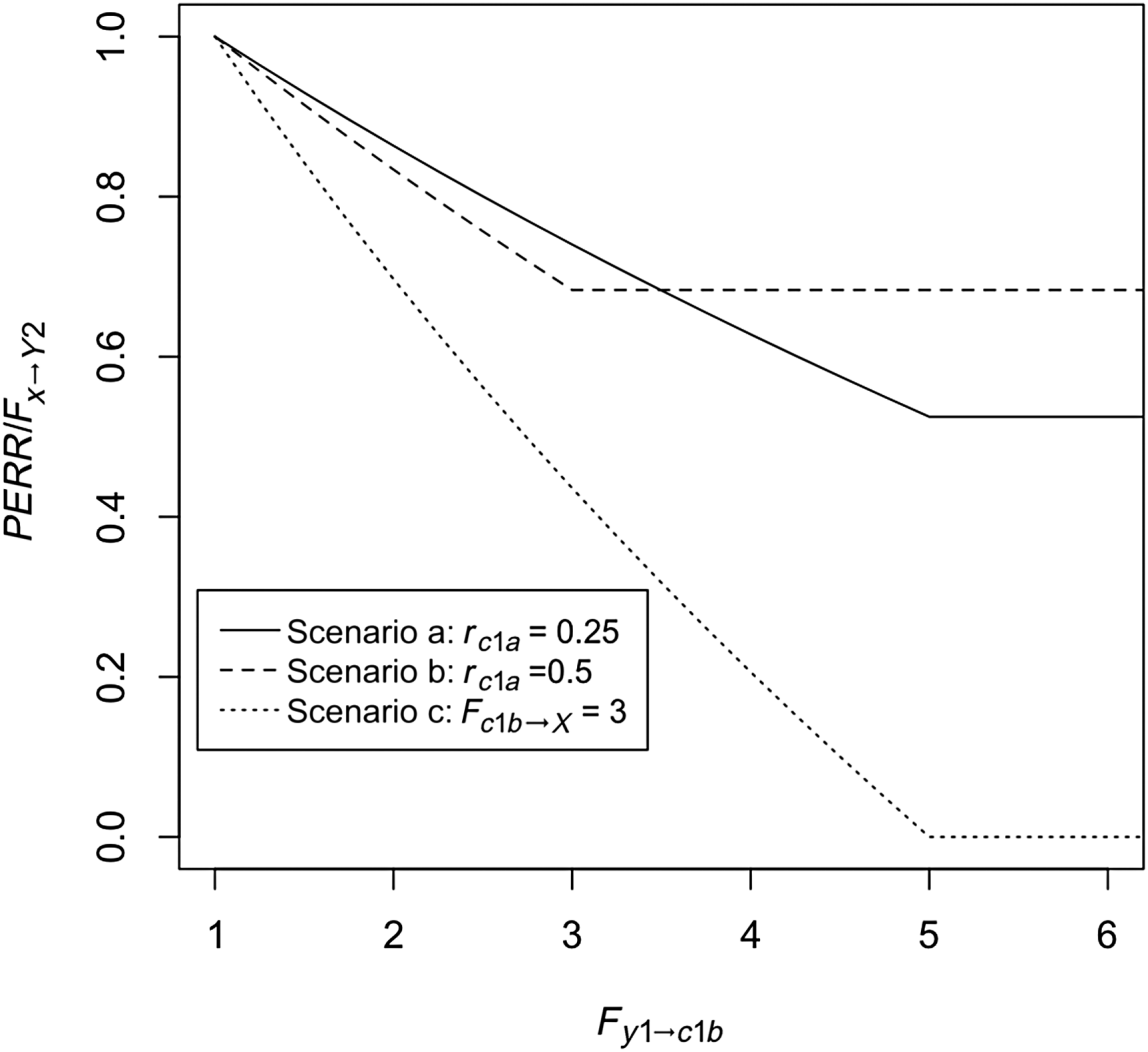
Performance of the prior event rate ratio (PERR) estimator when the directionality of the causal relationship between baseline confounder and *Y*_1_ is reversed, ie, *F*_*c*1*a* → *Y*1_ = 1 (presence of *c*_1*a*_ does not affect probability that *y*_1_ is present), *F*_*y*1 → *C*1*b*_ > 1 (presence of *y*_1_ increases the probability that *c*_1*b*_ is present), and as above, *c*_1*b*_ = *c*_1*a*_. Scenario a: *r*_*c*1*a*_ = 0.25. Scenario b: *r*_*c*1*a*_ = 0.5. Scenario c: *r*_*c*1*a*_ = 0.25, *F*_*c*1*b* → *X*_ is increased from 1.5 to 3

**Figure 7:**
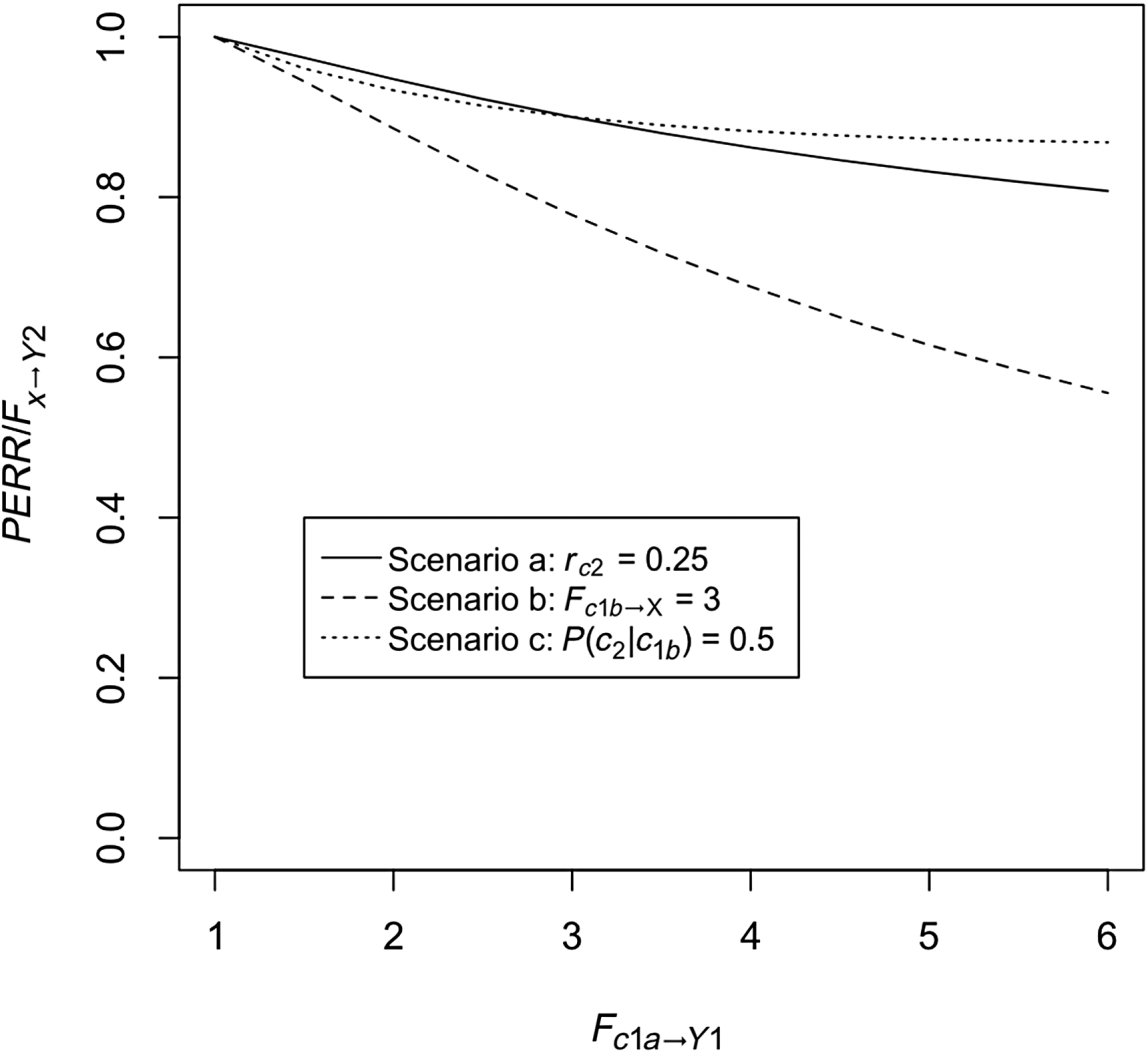
Performance of the prior event rate ratio (PERR) estimator when the value of *C*_2_ is allowed to differ from the value of *C*_1*b*_ (in all cases *C*_1*b*_ = *C*_1*a*_). Scenario a: *r*_*c*2_ = *r*_*c*1*a*_ = 0.25 and *F*_*c*1*b* → *C*2_ = 0.25^−1^ = 4, so that *P*(*c*_2_| *c*_1*b*_) = 1, $P\left(c_{2}|\bar{c_{1b}}\right)=0.25$ (ie, probability to gain confounder is 0.25). Scenario b: As in Scenario a but with *F*_*c*1*b* → *X*_ increased from 1.5 to 3. Scenario c: $P\left(c_{2}|c_{1b}\right)=0.5,P\left(c_{2}|\bar{c_{1b}}\right)=0$ (ie, probability of losing confounder is 0.5)

1. Unobserved confounder effect is present and affects the baseline and observation period event probability equally (ie, *F*_*c*2 → *Y*2_ = *F*_*c*1*a* → *Y*1_ ≠ 1) and also affects the probability of treatment (ie, *F*_*c*1*b* → *X*_ ≠ 1), while *F*_*y*1 → *X*_ is fixed at 1. Baseline period events do not affect the probability that the confounder is present (ie, *F*_*y*1 → *C*1*b*_ = 1). Figure 2 shows *RR*_*obs*_, the PERR, and true effect *F*_*x* → *Y*2_ as a function of *F*_*c*1*a* → *Y*1_. As we can see, even though *RR*_*obs*_ diverges from the true effect, the PERR is exactly equal to the true effect.
2. Retaining the effect of the unobserved confounder, we allow *F*_*y*1 → *Y*2_, the effect of the occurrence of a baseline event on the likelihood of an observation period event, to vary. Results are shown in Figure 3. As can be seen, increasing *F*_*y*1 → *Y*2_ above 1 makes the PERR progressively more pessimistic (ie, *PERR*/*F*_*x* → *Y*2_ increases above 1), though the effect is relatively weak and bounded.
3. We allow *F*_*c*2 → *Y*2_ to differ from *F*_*c*1*a* → *Y*1_. Results are shown in Figure 4. When *F*_*c*1*a* → *Y*1_ < *F*_*c*2 → *Y*2_, (ie, the effect of the confounder on likelihood of an event is weaker in the baseline period than in the observation period), the PERR is pessimistic; when the converse is true, *F*_*c*1*a* → *Y*1_ > *F*_*c* → *Y*2_, then the PERR is overoptimistic. As we will see later (Section 5 and Appendix A), one reason that these two values may differ is if the effect of treatment *x* in an individual changes depending on whether the confounder is present or absent.
4. We allow *F*_*y*1 → *X*_ to differ from 1; see Figure 5. Above 1, even modest values of *F*_*y*1 → *X*_ suffice to make the PERR substantially overoptimistic. Conversely, as *F*_*y*1 → *X*_ is decreased below 1, PERR rapidly becomes more pessimistic. Note that *F*_*y*1 → *X*_ cannot exceed $r_{X}^{-1}$, otherwise *P*(*X* = *x*) would exceed 1 for individuals having a baseline event *y*_1_.
5. We reverse the directionality of the baseline period relationship between confounder and event: while a pre‐existing confounder *c*_1_ does not affect the probability of a baseline event *y*_1_, an individual without pre‐existing *c*_1_ may develop *c*_1_ as a result of experiencing *y*_1_. Figure 6 shows that, as the strength of the association *F*_*y*1 → *C*1*b*_ increases, PERR becomes increasingly overoptimistic. Depending on the pre‐existing confounder incidence rate *r*_*c*1*a*_, and on *F*_*c*1*b* → *X*_, *PERR*/*F*_*x* → *Y*2_ may or may not have a lower limit >0.
6. We allow for the possibility that an individual either loses or gains the confounder between baseline and observation period. We see from Figure 7 that both scenarios act to make PERR overoptimistic; by how much depends on *F*_*c*1*a* → *Y*1_ and *F*_*c*1*b* → *X*_.

We thus see that the PERR works just as intended as long as the association between confounder and events has the same strength in the baseline and observation periods. The more confounding differs between the periods, though, the poorer the PERR does as an estimator of the true effect. Whether the strength of confounding increases or decreases with time is important, because this determines whether the PERR overestimates or underestimates, respectively, the true effect of the intervention.

We also see that the PERR is overoptimistic when there is a causal connection directed from baseline period event *y*_1_ to treatment *x*, either directly or via confounder *C*_1*b*_. Finally, PERR is overoptimistic when individuals are able to either gain or lose the confounder over the course of the study period.

### 4.1 Probabilistic bias analysis

We have investigated ways in which using the PERR to control for unobserved confounding can fail. However, if one has sufficient information to be able to place limits on the strengths of the causal associations among the study subjects' set of attributes {*C*_1*a*_, *Y*_1_, *C*_1*b*_, *X*, *C*_2_, *Y*_2_}, and the incidence rates of the unobserved confounders, one may still be able to obtain useful constraints on the true treatment effect via PBA.^11^

The approach is straightforward: one chooses prior probability distributions for the incidence rates of the unobserved confounder, and for each of the factors *F* governing the associations among the attributes, except for the treatment effect itself, *F*_*x* → *Y*2_. One then performs iterations of drawing a set of values from these distributions. For each set, one computes the value of *F*_*x* → *Y*2_ needed to realize the observed rates *R*(*X*), $R\left(Y_{1}|X\right)$, $R\left(Y_{1}|\bar{X}\right)$, $R\left(Y_{2}|X\right)$, and $R\left(Y_{2}|\bar{X}\right)$. In this way, one obtains a posterior distribution of possible values of the true treatment effect. The number of iterations should be sufficiently large that increasing it further does not appreciably change the shape of the posterior distribution. Here, we implement all of the above in R and perform 50 000 iterations for each scenario.

## 5 APPLICATION TO AN OBSERVATIONAL STUDY

We apply our method to a study of HD influenza vaccine effectiveness, relative to SD vaccine, against influenza and influenza‐associated outcomes that was conducted within the VA patient population by Young‐Xu et al.^9^ In this study, a difference in the rate of hospitalization for pneumonia and influenza (P&I; ICD‐9 codes 480‐488) in the HD and SD arms was found in the baseline period even after matching on patient comorbidities, suggesting residual confounding by indication. This was addressed through use of PERR adjustment. We will apply the above PBA methodology to assess the robustness of this approach in this particular study.

For the baseline period, the study reported event rates (in units of events per 10 000 person‐weeks) of *R*_*HD*,*base*_ = 3.24 and *R*_*SD*,*base*_ = 2.1. For the observation period, the rates were *R*_*HD*,*obs*_ = 3.45, *R*_*SD*,*obs*_ = 2.98. Thus, the RRs of HD versus SD arms in the baseline and observation periods were *RR*_*base*_ = 1.54 and *RR*_*obs*_ = 1.16, respectively. This made the unadjusted relative effectiveness *rVE*_*HD*,*obs*_ = 1 − *RR*_*obs*_ =  − 16%, suggesting (contrary to evidence from its RCT^12^) that the HD vaccine was less effective than SD. However, the fact that the RR in the baseline period differed significantly from 1 (*RR*_*base*_ = 1.54) suggested confounding by indication. The PERR estimate of the RR was

$$RR_{\text{PERR}}=\frac{RR_{\text{obs}}}{RR_{\text{base}}}=0.75,$$

for an HD to SD relative effectiveness

$$\text{rV}E_{\text{HD},\text{PERR}}=100\%\cdot \left(1-RR_{\text{perr}}\right)=25\left(2;43\right)\%,$$

where the confidence interval was obtained using the assumption of Poisson‐distributed events. We apply the causal diagram of Figure 1 to this study, with the following interpretation: starting in the baseline period, an unobserved confounder *C* that is present in part of the patient population—we can think of it as frailty not captured in the patients' medical records—potentially causes one or more of the following:

1. an elevated likelihood of hospitalization for pneumonia/influenza during the baseline period;
2. a modified likelihood of receiving HD rather than SD vaccine via confounding by indication: the presence of *C*_1_ increases the likelihood that the healthcare provider (HCP) will identify the patient as being at elevated risk, and this may then affect the HCP's decision whether to prescribe SD or HD;
3. an elevated likelihood that the patient will also possess the confounder in the observation period, ie, *C*_2_ = *c*_2_ (a frail patient is likely to remain frail);
4. if the confounder does carry over into the observation period, then an elevated likelihood of hospitalization for pneumonia/influenza during the observation period; and
5. a reduced immune response to vaccination, resulting in a reduction in the protective effect derived from both HD and SD.

Point 5 requires further explanation. It has been shown that frailty can substantially reduce the effectiveness of influenza vaccine against influenza‐associated hospitalization.^13^ Furthermore, it has been shown that the relative efficacy of HD versus SD does not vary significantly between frail and nonfrail individuals.^14^ We thus make the assumption that frail individuals receive a weakened vaccine effect, such that the RR due to vaccination is multiplied by a factor *f* > 1 for both HD and SD vaccination. It can be shown (see Appendix A) that this is equivalent to increasing *F*_*c*2 → *Y*2_ by the same factor, which, in turn, suggests that *F*_*c*2 → *Y*2_ > *F*_*c*1*a* → *Y*1_. In our PBA in the following (and in some of the additional analyses in Appendix B), we use the parameterization *F*_*c* → *Y*2_ = *fF*_*c* → *Y*1_. From Section 4, we know that, when *f* ≥ 1, this puts us in the regime of a pessimistic PERR, ie, one that will *underestimate* the relative effectiveness of HD, all other things being equal.

In the context of this study, the edge directed from *Y*_1_ to *X* in Figure 1 represents the possibility that the HCP's choice of vaccine is directly influenced by whether or not a patient was hospitalized for pneumonia/influenza during the baseline period. P&I hospitalization can also influence vaccine choice by the causal path from *Y*_1_ to *C*_1*b*_ to *X*: hospitalization may cause frailty (instead of/in addition to the other way around), and the presence of this newly acquired frailty may then influence vaccine choice. As we saw in Section 4, both the direct and indirect path can cause the PERR to be overoptimistic.

To investigate the possibility of a direct link from *Y*_1_ to *X*, we conducted an interview study among nurse infection preventionists in 25 Veterans Health Administration (VHA) facilities to gain understanding of the decision‐making process governing the selection of HD versus SD for a given patient within VA facilities. The study is described in Appendix C. In no case was previous hospitalization for pneumonia/influenza reported as a criterion for preferentially administering HD. On the contrary, in two (8%) of the facilities, recent hospitalization for pneumonia was reported as a possible contra‐indication for HD administration. This suggests that *F*_*y*1 → *X*_ ≤ 1.

We conduct our probabilistic bias analyses by performing Monte Carlo simulations consisting of 50 000 realizations each (some of these are discarded due to having infeasible combinations of inputs). Each realization proceeds via a “draw and adjust” procedure, as follows.

1) A set of observed values for $R\left(y_{1}|x\right)$, $R\left(y_{1}|\bar{x}\right)$, $R\left(y_{2}|x\right)$, and $R\left(y_{1}|\bar{x}\right)$ is drawn from the results of the study of Young‐Xu et al, assuming, as they do, that event counts are Poisson‐distributed.
2) All DAG parameters apart from *F*_*c*1 → *X*_, *r*_*x*_, *F*_*x* → *Y*2_ and *r*_*y*2_ are drawn from their respective prior distributions, and any required relationships among parameters are imposed.
3) *F*_*c*1*b* → *X*_ is chosen such that *R*(*y*_1_| *x*) is reproduced.
4) *r*_*x*_ is chosen such that *R*(*x*), the study HD vaccination rate, is reproduced.
5) *F*_*x* → *Y*2_ is chosen such that $RR_{\text{obs}}=\frac{R\left(y_{2}|x\right)}{R\left(y_{2}|\bar{x}\right)}$ is reproduced.

The output of each realization is the posterior distribution of *F*_*x* → *Y*2_, the true treatment effect, where *rVE*_*HD*,*true*_ = 100 %  · (1 − *F*_*x* → *Y*2_).

**Figure B1:**
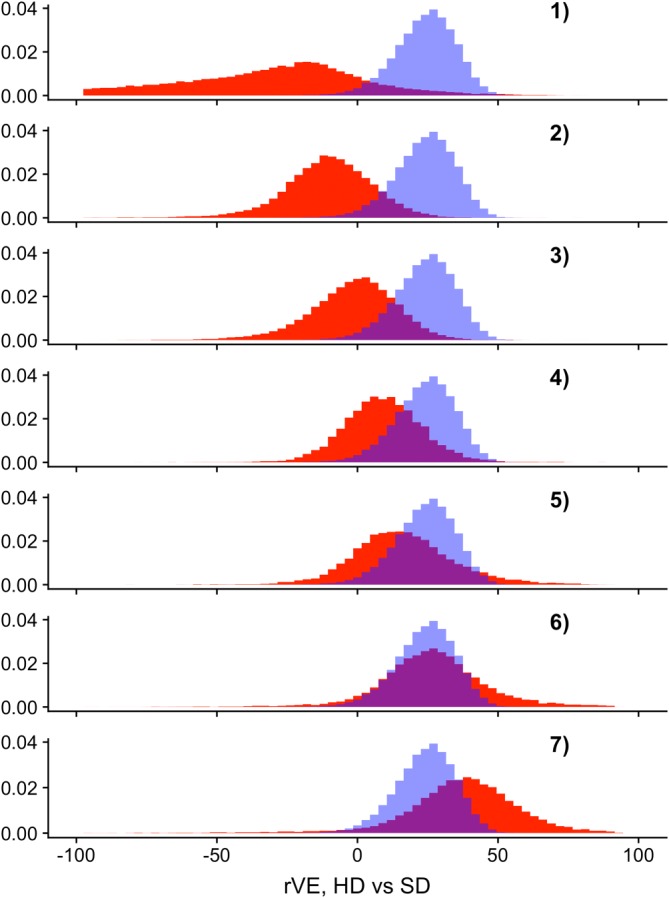
Results of probabilistic bias analysis to assess the true relative effectiveness of high‐dose (HD) versus standard‐dose (SD) influenza vaccine, *rVE*_*HD*_, for the scenarios in Table B1 [Colour figure can be viewed at http://wileyonlinelibrary.com]

We choose uniform distributions for all input parameters. In Appendix B, we examine the effect of applying progressively more constraints on the input parameter ranges and relationships among them (Table B1). Corresponding posterior distributions of *rVE*_*HD*,*true*_ are shown in Figure B1. As can be seen, under minimal assumptions (Analysis 1), PERR is very likely to significantly overestimate *rVE*_*HD*_. The distribution of *rVE*_*HD*,*true*_ becomes tighter and/or moves further to the right in each successive analysis, and for Analyses 6 and 7, PERR is more likely to underestimate than overestimate the true effect size.

**Figure 8:**
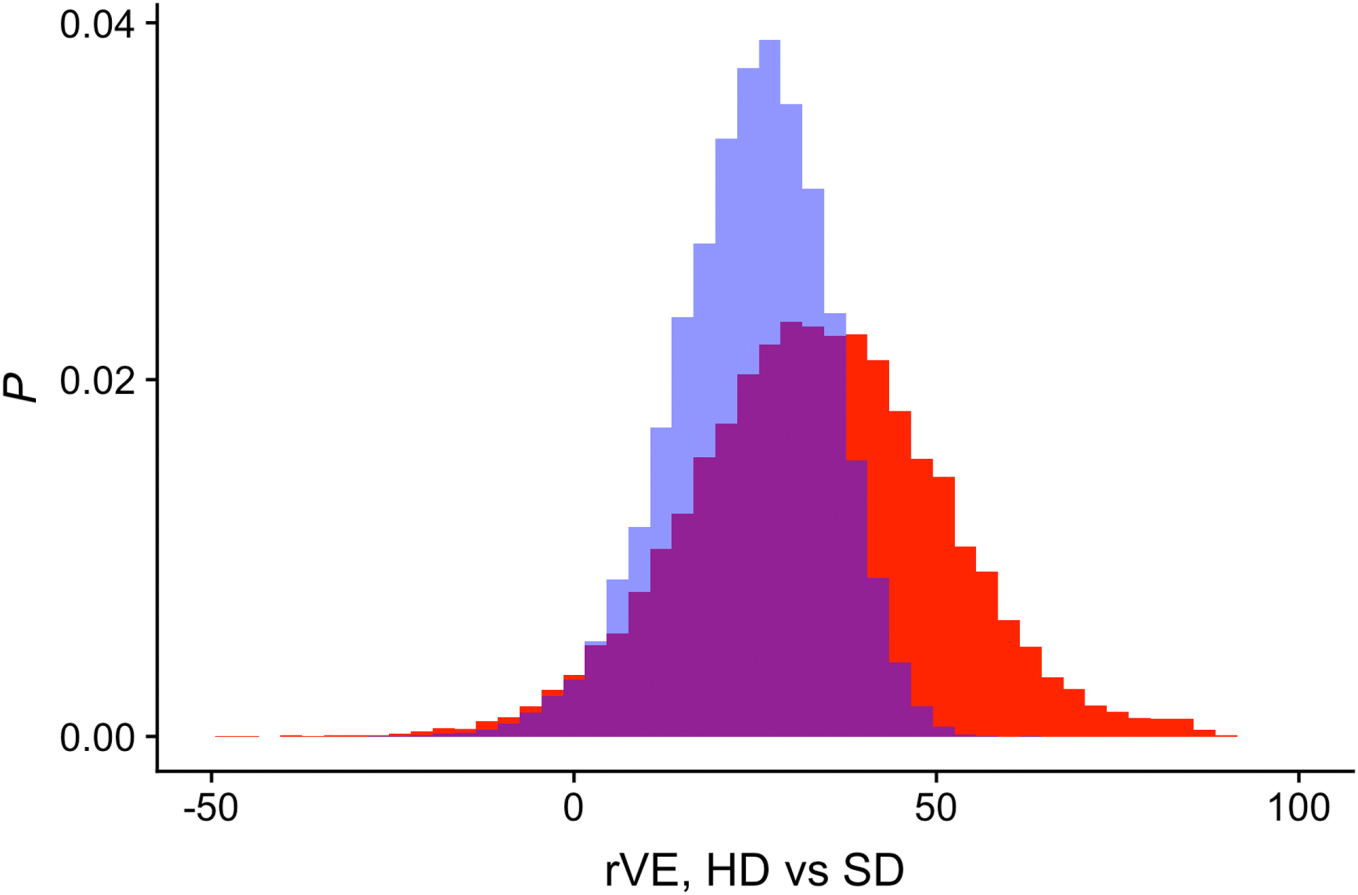
Output of the probabilistic bias analysis (inputs described in Table 1): posterior distribution of the true treatment effect (red), with the prior event rate ratio estimate of Young‐Xu et al^9^ (blue) shown for comparison. HD, high dose; SD, standard dose [Colour figure can be viewed at http://wileyonlinelibrary.com]

We now seek to identify specific constraints appropriate for this study. Table 1 gives a set of constraints, informed by published literature, on the relationship between hospitalization and frailty state transitions (see the work of Gill et al^15^) and on hospitalization rates, including for P&I, of high‐risk and low‐risk individuals (see the work of Mullooly et al^16^). In using the latter, we assume that the RR of P&I hospitalization of high‐risk versus low‐risk subjects can be used as a proxy for that of frail versus nonfrail subjects. The results of the PBA are given in Table 2, shown graphically in Figure 8, and compared to the PERR estimate in the study of Young‐Xu et al.^9^ Both distributions have a similar lower 95% CI bound, but both the median and upper bound of the PBA posterior distribution are higher than those of the PERR estimate. In Table 1, we compare the distributions using two different metrics: the common‐language effect size (CLES)^17^ and the two‐sample Hodges‐Lehmann estimator^18^ for the difference between two populations. The CLES gives the probability that a sample drawn from the distribution of *rVE*_*HD*,*PERR*_ is greater than a sample drawn from the distribution of *rVE*_*HD*,*true*_. In other words, it gives the probability that PERR overestimates the true effect size. The two‐sample Hodges‐Lehmann estimator is a nonparametric estimator of the median difference between a pair of samples drawn from the two distributions. Both comparisons suggest that the PERR estimate in the study of Young‐Xu et al is more likely to have underestimated than overestimated the true relative effectiveness of HD vaccine.

**Table 1:** Input parameter ranges for probabilistic bias analysis (PBA) of the study of Young‐Xu et al,^9^ together with literature sources/rationales

	PBA input parameters					
	RR of direct	Probability that	Probability that	Relationship	RR of effect of	Probability that
	effect of baseline	nonfrail subject	subject frail in	between frailty	baseline	subject nonfrail
	hospitalization	becomes frail	baseline period	effect on	hospitalization on	in baseline period
	on probability of	within baseline	becomes	hospitalization in	baseline frailty:	becomes frail in
	HD receipt (*F*_*y*1 → *X*_)	period (*r*_*c*1*b*_)	nonfrail in	observation and	(*F*_*y*1 → *C*1*b*_)	observation
			observation	baseline period:		period (*r*_*c*2_)
			period ( $P(\bar{c_{2}}|c_{1b}$))	( $f\equiv \frac{F_{c2\to Y2}}{F_{c0\to Y1}}$)		
						
Constraints	[0.9, 1.1]	[0.011, 0.024]	[0.0006, 0.0036]	[1, 10]	[1.06; 1.66]	[0.011; 0.024]
Source	Veterans Health	15	15	Appendix A, 13, 14	15	15
	Administration					
	Survey study,					
	Appendix C					
Comments	Survey	6‐month	6‐month	*f* > 1 follows	Hazard ratio of	6‐month
	(Appendix C)	incidence rate of	incidence rate of	from assumption	transition rate	incidence rate of
	suggests *F*_*y*1 → *X*_ ≤ 1; we	transition from	transition from	that frail	from frail to nonfrail	transition from
	conservatively	nonfrail to frail	frail to nonfrail	individuals derive	state after	nonfrail to frail
	assume RR that varies	state (their Table 1)	state (their Table 1)	less protection	hospitalization,	state (their Table 1)
	by +/− 10% about 1			from vaccine	vs. no intervening	
				than nonfrail	hospitalization	
					(their Table 2)	

**Table 2:** Output of the probabilistic bias analysis (inputs described in Table 1): posterior distribution of the true treatment effect, and comparison to the prior event rate ratio (PERR) estimate in the study of Young‐Xu et al^9^

***rVE***_***HD,true***_(%)	**Comparison to** ***rVE***_***HD*,*PERR***_ **= 25**(**2;42**)%	
	Common‐language effect size:	Two‐sample Hodges‐Lehmann
	probability that PERR **overestimates** true	estimate of median difference (%) (a
	effect, ie, *P*(*rVE*_*HD*,*PERR*_ > *rVE*_*HD*,*true*_)	**positive** value is a PERR **overestimate**)
34 (0; 42)	0.28	−11

## 6 DISCUSSION

In multiple studies,^1^, ^2^, ^3^ PERR adjustment has been demonstrated to perform well in controlling for bias due to unmeasured confounding. The method is also appealing due to its relative simplicity, both conceptually and in implementation. However, it has been shown that this method may in some cases increase rather than decrease bias.^5^ We further explored PERR performance and used Bayesian network calculations applied to the causal diagram representation of a previously published observational study of the relative effectiveness of HD versus SD influenza vaccine in the US VA patient population. Using PBA, we showed that, in applying the PERR estimator to control for unmeasured confounding, this particular study is more likely to have underestimated rather than overestimated the true effect size.

This is not to argue that the PERR should be categorically discarded in favor of PBA on Bayesian networks. Under appropriate conditions, the PERR alone will suffice. With reference to the causal diagram of Figure 1, the PERR can safely be used if all of the following apply:

(i) A direct causal association between baseline period event *Y*_1_ and treatment *X* can be ruled out. Such an association can masquerade as an unmeasured confounder, and naïve application of the PERR in this case acts to increase rather than decrease bias.
(ii) There is no variation in the strength of the confounder effect between baseline and observation period.
(iii) Individuals can neither gain nor lose the confounder during the study period.
(iv) A bidirectional relationship between confounder and baseline‐period event can be ruled out; that is, presence of a baseline event does not affect the probability that the confounder is present.
(v) No direct causal association between baseline period event *Y*_1_ and observation period event *Y*_2_ exists. In practice, this is the least critical constraint, since the effect of such an association on the accuracy of the PERR estimator is modest and bounded.

If (i) or (ii) is violated, but one knows the directionality of the relationship, then, depending on the study question, the PERR may still be able to provide a useful constraint on the treatment effect.

This work is subject to a number of limitations. To begin with, although the causal diagram we use is relatively generic, it by no means constitutes an adequate description of every possible type of real‐world study. For example, loss of study subjects due to mortality or dropout is not taken into account. In our example of the study of Young‐Xu et al,^9^ no subjects were lost during the baseline period, and less than 3% were lost during the observation period (unpublished data). Uddin et al^5^ did consider loss of subjects and showed that this will cause the PERR to overestimate the treatment effect. For a loss rate of less than 5%, they showed underestimation by well below 1%. However, any study in which mortality/dropout plays a more significant role will need to add this effect into the model. Also, because PERR involves repeat measurements of the same population, correlation effects may be present. In the study of Young‐Xu et al, this issue, as well as the possibility of cluster effects at the VA facility level, was addressed by conducting a sensitivity analysis using generalized estimating equations. Even so, our analysis abstracts time dependence into simply a baseline and an observation period. Studies in which time evolution of subjects is more complex will need to utilize a correspondingly more complex DAG, for example, one in which baseline and observation are broken down into multiple subperiods. It should also be emphasized that, in our PBA re‐analysis of the study of Young‐Xu et al, the sources we use to constrain PBA inputs are derived from a targeted literature search, not an exhaustive literature review. More broadly, any analysis of this type can only be as valid as the DAG that informs it. The construction of a DAG ultimately relies on expert opinion, and no amount of expert opinion can guarantee that a causal link will not be misspecified or overlooked.

Despite the above limitations, our study offers an approach to understanding under what scenarios the PERR is likely to provide an unbiased estimate of the treatment, and a methodology to bound the bias when it is present.

## DISCLOSURES

E. Thommes, R. van Aalst, J. Lee, and A. Chit are employees of Sanofi Pasteur. S. Mahmud and Y. Young‐Xu have received funding from Sanofi Pasteur in the context of this and previous studies. J. Snider is an employee of and holds equity in Precision Health Economics, which has received consulting fees from Sanofi Pasteur for this and previous studies.

## DATA AVAILABILITY STATEMENT

The computer code used to generate the results of this study is included in the published article's supplementary information files.

## Supporting information

SIM_8435‐Suppl‐0001.zip

## APPENDIX A

With specific reference to the HD influenza study of Young‐Xu et al,^9^ let us rewrite Equation (9) in terms of the absolute (rather than relative) HD and SD effect and make things explicit by redefining the domain of *X* as *dom*(*X*) = [*HD*, *SD*]:

PY2=y2|C2,Y1,X=rY2·Fc→Y2,ifC2=c21,ifC2=c2‾·Fy1→Y2,ifY1=y11,ifY1=y1‾·FHD→Y2,ifX=HDFSD→Y2,ifX=SD.

Now, suppose further that individuals who are frail in the observation period (*C*_2_ = *c*_2_) receive an attenuated vaccine effect, such that *F*_*HD* → *Y*2_ and *F*_*SD* → *Y*2_ are both multiplied by a factor *f* > 1.

Thus, we have

PY2=y2|C2,Y1,X=ry2·Fc2→Y2,ifC2=c21,ifC2=c‾2·Fy1→Y2,ifY1=y11,ifY1=y1‾·f·FHD→Y2,ifX=HD,C2=c2FHD→Y2,ifX=HD,C2=c‾2f·FSD→Y2,ifX=SD,C2=c2FSD→Y2,ifX=SD,C2=c‾2,

which can be rewritten to absorb *f* into the term involving *F*_*c* → *Y*2_

PY2=y2|C2,Y1,X=ry2·f·Fc2→Y2,ifC2=c21,ifC2=c‾2·Fy1→Y2,ifY1=y11,ifY1=y1‾·FHD→Y2,ifX=HDFSD→Y2,ifX=SD.

Finally, if we define $r_{y2}^{\prime }=r_{y2}F_{\text{SD}\to Y2}$, we can write this as

PY2=y2|C2,Y1,X=ry2′·f·Fc2→Y2,ifC2=c21,ifC2=c‾2·Fy1→Y2,ifY1=y1,1ifY1=y1‾·FHD,rel,ifX=HD1,ifX=SD,

where

$$F_{\text{HD},\text{rel}}=\frac{F_{\text{HD}\to Y2,}}{F_{\text{SD}\to Y2}}$$

and

$$\text{rV}E_{\text{HD}}=100\%\cdot \left(1-F_{\text{HD},\text{rel}}\right).$$

If, in the absence of the attenuation, *F*_*c*2 → *Y*2_ = *F*_*c*1*a* → *Y*1_, then with attenuation factor *f* > 1,

(21) $$F_{c_{2}\to Y2}=f\cdot F_{c1a\to Y1}>F_{c1a\to Y1}.$$

## APPENDIX B

We begin with minimal assumptions about the input parameters (Analysis 1) and then consider a series of scenarios wherein we apply progressively more restrictive constraints on the prior ranges of the input parameters. All ranges are taken as uniform, and in all scenarios, we take *r*_*c*1*a*_ ∈ [0.25,0.75], *F*_*c*1*a* → *Y*1_ ∈ [1,10], and *F*_*y*1 → *Y*2_ ∈ [1,10]. For each analysis, we report the distributions of *rVE*_*HD*,*true*_ and compare it to *rVE*_*HD*,*PERR*_, the PERR estimate derived by Young‐Xu et al. Specifically, we wish to quantify to what degree the PERR over/underestimates the true treatment effect. We do so using two different metrics: the CLES^17^) and the two‐sample Hodges‐Lehmann estimator for the difference between two populations.^18^ The CLES gives the probability that a sample drawn from the distribution of *rVE*_*HD*,*PERR*_ is greater than a sample drawn from the distribution of *rVE*_*HD*,*true*_. In other words, it gives the probability that PERR overestimates the true effect size. The two‐sample Hodges‐Lehmann estimator is a nonparametric estimator of the median difference between a pair of samples drawn from the two distributions. The setups and results of the simulations are given in Table B1. Results are shown graphically in Figure B1.

**Table B1:** Setup and results of the set of seven probabilistic bias analyses (PBA) conducted on the Veterans Health Administration study of Young‐Xu et al.^9^ Starting with minimal assumptions about the relationships among ***C***_**1*a***_, ***C***_**1*b***_, ***C***_**2**_, ***Y***_**1**_, ***X***, and ***Y***_**2**_, we add assumptions (described in the leftmost column) one by one and apply the corresponding constraints on the input parameters. Cells corresponding to tightened constraints are shown in light gray, and darker gray for the one instance of a constraint further tightened

	PBA input parameters					Results		
**Assumptions**	RR of direct	Probability	Relationship	RR of effect	Probability	*rVE*_*HD*,*true*_	Comparison to *rVE*_*HD*,*PERR*_=	
	effect of	that frail	between	of baseline	that nonfrail	(%)	25(2; 43)%	
	baseline	subject	frailty effect	hospitalization	subject		Common	Two‐sample
	hospitalization	(baseline)	on	on baseline	(baseline)		language effect	Hodges‐
	on probability	becomes	hospitalization	frailty:	becomes		size (CLES):	Lehmann
	of HD receipt	nonfrail	in observation	(*F*_*y*1 → *C*1,*b*_)	frail		probability that	estimate of
	(*F*_*y*1 → *X*_)	(observation)	and baseline		(observation)		PERR	median
		( $P\left(\bar{c_{2}}|c_{1b}\right)$)	period:		scaled by		overestimates	difference (%)
			( $f\equiv \frac{F_{c2\to Y2}}{F_{c0\to Y1}}$)		baseline		true effect, ie,	(**positive** values
					frailty rate		*P*(*rVE*_*HD*,*PERR*_	are PERR
					( $F_{\text{new}}\equiv \frac{P_{\text{frail}}}{r_{c1a}}$)		>*rVE*_*HD*,*true*_)	**overestimates**)
1) Minimal assumptions	[0.1, 10]	[0, 1]	no relationship	[1, 10]	[0, 1]	−31(−184; 30)	0.957	57
2) No direct effect of	1	[0, 1]	no relationship	[1, 10]	[0, 1]	−10(−46; 19)	0.968	34
baseline hospitalization								
on probability of receiving HD								
3) Subjects do not recover	1	0	no relationship	[1, 10]	[0, 1]	0(−37; 26)	0.927	26
from frailty								
4) Frailty effect on	1	0	[1, 10]	[1, 10]	[0, 1]	9(−18; 37)	0.814	16
hospitalization is at least								
as strong in observation								
as baseline period								
5) In baseline period, frailty	1	0	[1, 10]	[1, *F*_*c*0 → *Y*1_]	[0, 1]	15(−17; 57)	0.682	9
effect on hospitalization								
is at least as strong as								
hospitalization								
effect on frailty								
6) Previously nonfrail	1	0	[1, 10]	[1, *F*_*c*0 → *Y*1_]	0	26(−12; 68)	0.459	−1
subjects do not								
become frail in								
observation period								
7) In baseline period,	1	0	[1, 10]	1	0	38(−13; 74)	0.245	−14
hospitalization								
has no effect on								
frailty								

**Table C1:** Study participants contacted and interviewed (data collected) by high dose (HD) influenza vaccine group

Proportion of		Contacted		Data Collected	
HD vaccine	Selected VHA	Medical	Interviewees	Medical	Interviewees
administered*	Medical Centers	Centers	(potential)	Centers	(consented)
**A: 0%‐4%**	41	19	36	6	9
**B: 5%‐29%**	17	17	37	8	9
**C: 30%‐69%**	12	12	27	6	8
**D: 70%‐100%**	12	12	25	5	8
**TOTAL**	**82**	**60**	**118**	**25**	**34**

* During the 2014‐2015 season; VHA: Veterans Health Administration.

**Table C2:** High dose (HD) influenza vaccine quantities ordered and used during 2015‐2016 and 2016‐2017 among study participants (Veterans Health Administration facilities) by group

Proportion of	Season 1: 2015‐2016**				Season 2: 2016‐2017**				Season 2‐Season 1 Difference***		
HD vaccine	N	Ordered	Used	% Used	N	Ordered	Used	% Used	N	Ordered	Used
**administered** *											
**A: 0%‐4%**	6	16 150	12 753	**79.0%**	6	29 400	24 208	**82.3%**	6	13 250 (58.2%)	11 455 (62.0%)
**B: 5%‐29%**	5	29 100	26 806	**92.1%**	3	10 050	9950	**99.0%**	4	−50 (−0.5%)	3644 (13.0%)
**C: 30%‐69%**	6	1120	1075	**96.0%**	6	18 000	16 371	**91.0%**	4	12 880 (170.4%)	12 496 (170.6%)
**D: 70%‐100%**	3	23 000	19 993	**86.9%**	4	42 290	41 356	**97.8%**	3	3000 (12.2%)	5073 (22.5%)
**TOTAL**	**20**	**69 370**	**60 627**	**87.4%**	**19**	**99 740**	**91 885**	**92.1%**	**17**	**29 080 (44.1%)**	**32 668 (42.7%)**

* During the 2014‐2015 season.

** Includes those facilities reporting ordered and used amounts.

*** Includes those facilities reporting ordered and/or used amounts for both seasons.

## APPENDIX C

### INTERVIEW SURVEY: HIGH‐DOSE VACCINE ALLOCATION IN THE VETERANS HEALTH ADMINISTRATION

**BACKGROUND**

The objective of this survey study was to understand the contributing factors to the variations in the HD influenza vaccine coverage among VHA facilities and to gain insight into the decision‐making process for determining eligibility for and access to HD vaccine.

**METHODS**

We used HD vaccine proportion to measure facilities' HD vaccine coverage. The numerator for the measure was calculated as the number of patients aged 65 and older who received the HD influenza vaccine during the 2014‐2015 influenza season and the denominator was the total number of patients 65 and older who received either the HD or SD influenza vaccine during the 2014‐2015 influenza season. Administration of the flu vaccine, as well as the vaccine type, were determined by CPT codes (HD: 90662, SD: 90655‐90659, Q2034‐Q2039) from VHA EMRs. VHA medical centers were stratified into four groups according to HD vaccine proportion: 0%‐4%, 5%‐29%, 30%‐69%, and 70%‐100%.

Initially, 10 facilities were randomly selected from each of the four HD vaccine proportion groups. For each site, we randomly selected and invited one nurse infection preventionist at a time to participate in a 10‐15‐minute telephone interview. If the interviewee was unable to provide all requested information at the time of the interview, follow‐up interviews were performed with the individual, and/or additional facility personnel, such as other infection preventionists or pharmacists. If no response to the initial invitation(s) was received within a specified amount of time, another facility from within the same HD vaccine proportion group was selected at random, and the process repeated.

The content of the interview focused on the two most recent influenza seasons: 2015‐2016 and 2016‐2017. Twenty‐two questions were organized into the following four areas.

1. HD vaccination policy (n = 7)
2. Defining criteria for a high‐risk patient (n = 6)
3. Contra‐indication for the HD vaccine (n = 3)
4. Quantity of HD vaccine (n = 6)

Responses were summarized as frequencies at the HD proportion group level. Where possible, numbers were imputed based on the type of response provided. For example, for the purpose of calculating percentages of HD vaccine used, we were able to ascertain the number ordered and the number used if percentages and at least one quantity were reported. Due to the small sample size and descriptive nature of this study, no statistical tests were performed.

**RESULTS**

Data were collected from 34 employees at 25 facilities (Table C1). Sixteen (64%) of the responding facilities provided complete information requested in the survey.

*HD vaccine ordered and used*

Nine (36%) facilities provided partial data for either the quantity of HD vaccine ordered or percentage used, or were able to provide the information for one season only. The ordered, used and percentage HD used varied greatly across the four groups and within each group (Table C2). Only 8 out of 20 facilities (40%) and 10 out of 19 (53%) reported utilizing 100% of the ordered HD in 2015‐2016 and 2016‐2017, respectively. Of the 17 facilities that reported ordered HD quantities for both seasons, 10 (59%) increased, 1 (6%) decreased, and 6 (35%) did not alter the amount ordered for the following 2016‐2017 influenza season as compared to 2015‐2016. Also, 40% (N = 10) increased the amount of HD ordered for the most recent season, 2017‐2018, as compared to 2015‐2016 and 2016‐2017.

*Decision making process for HD vaccine acquisition*

Primary decision‐makers for the quantity of seasonal HD supply reported were similar across the four groups as pharmacy and/or a multidisciplinary committee consisting of representatives from various specialties, most commonly Infection Control and Infectious Diseases (Table C3). In seven (28%) facilities, pharmacy was a sole decision‐maker for determining the amount of HD to be ordered for a season.

**Table C3:** Mechanisms of acquiring high dose (HD) influenza vaccine among study participants (Veterans Health Administration facilities) for the 2015‐2016 and 2017‐2018 seasons by group

Characteristic		Proportion of HD vaccine administered during the 2014‐2015 season				
		A: 0%‐4%	B: 5%‐29%	C: 30%‐69%	D: 70%‐100%	Total
						
		N = 6	N = 8	N = 6	N = 5	N = 25
**HD quantity decision makers**	Pharmacy	5 (83.3%)	6 (75.0%)	4 (66.7%)	3 (60.0%)	**18 (72.0%)**
	Infection Control	1 (16.7%)	2 (25.0%)	1 (16.7%)	1 (20.0%)	**5 (20.0%)**
	Multidisciplinary committee	2 (33.3%)	2 (25.0%)	3 (50.0%)	3 (60.0%)	**10 (40.0%)**
	Department Chiefs/					
	Individual Providers	2 (33.3%)	2 (25.0%)	2 (33.3%)	1 (20.0%)	**7 (28.0%)**
**Barriers to ordering**	Cost	1 (16.7%)	1 (12.5%)	0	0	**2 (8.0%)**
	Lack of evidence	0	3 (37.5%)	1 (16.4%)	1 (20.0%)	**5 (20.0%)**
	Prior waste	1 (16.7%)	1 (12.5%)	0	0	**2 (8.0%)**
**Shortage in at least one season?**		2 (33.3%)	3 (42.9%)	3 (50.0%)	3 (60.0%)	**10 (40.0%)**
**Unused vaccine in at least one season?**		4 (66.7%)	2 (28.6%)	2 (33.3%)	3 (60.0%)	**11 (44.0%)**
**Reason for unused vaccine**	Staff unaware of supply	2 (33.3%)	0	1 (16.7%)	0	**3 (12.0%)**
	Adherence to administration	1 (16.7)	0	0	1 (20.0%)	**2 (8.0%)**
	criteria					
	Other (ie, extra supply					
	received from other facility,					
	lack of demand, low					
	vaccination levels, and poor					
	documentation)	1 (16.7%)	1 (12.5%)	1 (16.7%)	1 (20.0%)	**4 (16.0%)**
**Ordered more for 2017/2018 season**		3 (60.0%)	3 (42.9%)	1 (25.0%)	3 (60.0%)	**10 (40.0%)**

Overall, 10 (40%) facilities reported a shortage or inability to meet the demand for HD vaccine, and, for some, this occurred despite reported unused HD vaccine at the end of the season (N = 3). In these instances, the leftover doses were attributed to strict adherence to the criteria for HD administration (N = 1), extra supply received from other facilities (N = 1), and for one, no explanation was offered. Of the 12 facilities that reported unused HD, the remaining 9 (75%) reported no shortage or unmet demand. Reasons for the surplus cited included staff unaware of HD availability (N = 3), strict adherence to the criteria for HD administration (N = 1), lack of demand (N = 1), low vaccination rates overall (N = 1), possibly poor documentation of administered HD (N = 1), and for two, no explanation was offered.

The cost of HD vaccine (N = 2), lack of conclusive evidence (N = 5), and unused doses of HD in the prior season (N = 2) were the reported barriers to increasing HD supply for the future seasons. A common theme among the surveyed infections preventions and pharmacists was the perception of a lack of clear guidance from the Centers for Disease Control and Prevention (CDC) or in the scientific literature regarding administration of HD vaccine.

*Criteria, policies, and practices for HD administration*

Twenty (80%) of surveyed facilities have a standing nursing order for HD administration and the use of such a practice varied from 100% for Group A to 67% for Group C (Table C4). Similarly, a majority or 18 (72%) have a facility‐wide policy on HD encompassing: age alone (N = 13); a combination of age and presence of certain comorbidities (N = 4); or positive HIV status (N = 1). Ten (40%) facilities have department‐specific policies or practices for: Home‐Based Primary Care (N = 2), acute care (N = 3), and Community Living Center (CLC)‐residing (N = 8), patients, as well as geriatric (N = 1), Infectious Diseases (N = 1), and HIV (N = 1) clinics. Provider discretion for HD administration on an individual basis despite the presence of facility‐wide or department‐specific policies was reported by 20 (80%) of facilities, and, furthermore, for 18 (72%), respondents reported that providers specifically target patients they determine to be at high‐risk not otherwise covered in an existing policy.

**Table C4:** Policies and practices for distributing high dose (HD) influenza vaccine among study participants (Veterans Health Administration facilities) for the 2015‐2016 and 2017‐2018 seasons by group

Characteristic		Proportion of HD vaccine administered during the 2014‐2015 season				
		A: 0%‐4%	B: 5%‐29%	C: 30%‐69%	D: 70%‐100%	Total
						
		N = 6	N = 8	N = 6	N = 5	N = 25
**Standard nursing order/protocol**		6 (100%)	6 (75.0%)	4 (66.7%)	4 (80.0%)	**20 (80.0%)**
	Applies?	6 (100%)	4 (50.0%)	3 (50.0%)	5 (100%)	**18 (72.0%)**
**Facility‐wide policy**	Age (65+)	6 (100%)	3 (37.5%)	3 (50.0%)	5 (100%)	**17 (68.0%)**
	Immuno‐					
	compromised/HIV	1 (16.7%)	3 (37.5%)	0	0	**4 (16.0%)**
	Chronic	1 (16.7%)	1 (12.5%)	1 (16.7%)	0	**3 (12.0%)**
**Department‐specific policy/practice**	Applies?	2 (33.3%)	2 (33.3%)	3 (50.0%)	0	**10 (40.0%)**
	CLC/HBPC	1 (16.7%)	5 (62.5%)	3 (50.0%)	0	**9 (36.0%)**
	Acute care	1 (16.7%)	2 (25.0%)	0	0	**3 (12.0%)**
	Specialty Clinics	0	3 (37.5%)	0	0	**3 (12.0%)**
**Provider discretion**	Applies?	5 (83.3%)	7 (87.5%)	5 (83.3%)	3 (60.0%)	**20 (80.0%)**
	MD	5 (83.3%)	7 (87.5%)	5 (83.3%)	3 (60.0%)	**20 (80.0%)**
	NP/PA	4 (66.7%)	7 (87.5%)	4 (66.7%)	3 (60.0%)	**18 (72.0%)**
	RN	2 (33.3%)	6 (75.0%)	1 (16.7%)	1 (20.0%)	**10 (40.0%)**
	LPN	0	1 (12.5%)	0	0	**1 (4.0%)**
**Target patients at high‐risk not covered by policies**		6 (100%)	6 (75.0%)	3 (50.0%)	3 (60.0%)	**18 (72.0%)**

Overall, 14 (56%) reported a facility‐wide policy, 6 (24%) department‐specific policies, and 4 (16%) a combination of both facility‐wide and department‐specific policies, while 1 (4%) reported relying solely on provider's discretion for administering HD in patients who are aged 65 years and older. All facilities in the highest HD proportion group (D) reported using a facility‐wide policy on the basis of age alone and no department‐specific policies; however, 60% (N = 3) of these reported that providers are able to use their discretion to target high‐risk patients if they do not meet the age criterion.

Defining high‐risk in the setting of HD vaccination varied from one facility to another: 22 (88%) consider advanced age (defined as 65+ (N = 20), 80+ (N = 1) or 85+ (N = 1)) to be a criterion, which aligns with the policies described above (Table C5). In 18 (72%) of these 22 facilities, meeting the age criterion alone was sufficient to be considered at high‐risk, whereas for four (16%), such a consideration necessitated a combination of advanced age and the presence of certain chronic conditions such as diabetes, chronic obstructive pulmonary disease, chronic heart disease, or immunocompromised status. In addition to these, other patient criteria reported included, but were not limited to positive HIV status, CLC residency or being seen in a high‐risk clinic.

**Table C5:** High‐risk patient criteria for high dose (HD) influenza vaccination administration among study participants (Veterans Health Administration facilities) for the 2015‐2016 and 2017‐2018 seasons by group

Characteristic		Proportion of HD vaccine administered during the 2014‐2015 season				
		A: 0%‐4%	B: 5%‐29%	C: 30%‐69%	D: 70%‐100%	Total
						
		N = 6	N = 8	N = 6	N = 5	N = 25
	65+	6 (100%)	6 (75.0%)	5 (83.3%)	5 (100%)	**22 (88.0%)**
**Age**	80+	1 (16.7%)	0	0	0	**1 (4.0%)**
	85+	0	0	0	1 (20.0%)	**1 (4.0%)**
**Home‐Based Primary Care patient**		2 (33.3%)	1 (12.5%)	1 (16.7%)	0	**4 (16.0%)**
**Inpatient** (acute care)		1 (16.7%)	1 (12.5%)		0	**2 (8.0%)**
**Community Living Center resident**		0	5 (62.5%)	3 (50.0%)	1 (20.0%)	**9 (36.0%)**
**Being seen inf high risk clinic** (eg, geriatrics, Infectious Diseases,		1 (16.7%)	2 (25.0%)	0	0	**3 (12.0%)**
HIV, pulmonary, nephrology, oncology, and rheumatology)						
**Immunocompromised**		3 (50.0%)	2 (25.0%)	3 (50.0%)	1 (20.0%)	**9 (36.0%)**
**HIV positive**		1 (16.7%)	2 (25.0%)	0	0	**3 (12.0%)**
	*HIV/AIDs w/low CD‐4 meter*	1 (16.7%)	0	0	0	**1 (4.0%)**
**Renopathic condition**		1 (16.7%)	1 (12.5%)	0	0	**2 (8.0%)**
	*on dialysis*	1 (16.7%)	0	0	0	**1 (4.0%)**
**Diabetes**		1 (16.7%)	2 (25.0%)	1 (16.7%)	0	**4 (16.0%)**
	*uncontrolled*	1 (16.7%)	0	0	0	**1 (4.0%)**
**Respiratory issues**		1 (16.7%)	1 (12.5%)	0	0	**2 (8.0%)**
	*Asthmatic*	1 (16.7%)	1 (12.5%)	0	0	**2 (8.0%)**
**Chronic pulmonary condition**		1 (16.7%)	1 (12.5%)	1 (16.7%)	1 (20.0%)	**4 (16.0%)**
	*Chronic obstructive pulmonary disease*	1 (16.7%)	1 (12.5%)	1 (16.7%)	0	**3 (12.0%)**
**Cardiovascular condition**		1 (16.7%)	1 (12.5%)	3 (50.0%)	0	**5 (20.0%)**
	*Heart disease*	0	0	2 (33.3%)	0	**2 (8.0%)**
	*Congestive heart failure*	1 (16.7%)	1 (12.5%)	1 (16.7%)	0	**3 (12.0%)**
**Neurological condition**		0	1 (12.5%)	0	0	**1 (4.0%)**
**Neuropathic condition**		0	1 (12.5%)	0	0	**1 (4.0%)**
**Other/unspecified chronic condition**		0	1 (12.5%)	0	0	**1 (4.0%)**
**Blood‐borne infection**		0	0	1 (16.7%)	0	**1 (4.0%)**
**Flu in the previous season**		0	0	1 (16.7%)	0	**1 (4.0%)**

*Contraindications for HD administration*

Eleven (44%) of the surveyed facilities reported contra‐indications for HD administration (Table C6). Two (8%) reported that a recent pneumonia hospitalization could be a reason to give a SD instead of HD vaccine to an individual. The most commonly reported reason for delay or avoidance of HD vaccination was prior history of allergic reaction to an influenza vaccine or its components (N = 8). Presence of fever was cited as the second most common contra‐indication (N = 5), followed by already immunized (N = 2), and history of Guillain‐Barre syndrome (N = 2).

**Table C6:** Contra‐indication criteria for high dose (HD) influenza vaccination administration among study participants (Veterans Health Administration facilities) for the 2015‐2016 and 2017‐2018 seasons by group

Characteristic	Proportion of HD vaccine administered during the 2014‐2015 season				
	A: 0%‐4%	B: 5%‐29%	C: 30%‐69%	D: 70%‐100%	Total
					
	N = 6	N = 8	N = 6	N = 5	N = 25
***Reported contra‐indication***	***3 (50.0%)***	***3 (37.5%)***	***3 (50.0%)***	***2 (40.0%)***	***11 (44.0%)***
History of allergic reaction to influenza vaccine or its components	3 (50.0%)	1 (12.5%)	2 (33.3%)	2 (40.0%)	**8 (32.0%)**
History of allergic reaction to eggs	1 (16.7%)	0	0	0	**1 (4.0%)**
Allergy to latex or thimerosal	0	1 (12.5%)	0	0	**1 (4.0%)**
Already immunized	1 (16.7%)	0	1 (16.7%)	0	**2 (8.0%)**
History of Guillain‐Barre syndrome	1 (16.7%)	0	0	1 (20.0%)	**2 (8.0%)**
Not feeling well	1 (16.7%)	0	0	0	**1 (4.0%)**
Moderate to severe acute illness	1 (16.7%)	0	1 (16.7%)	0	**2 (8.0%)**
Presence of fever	1 (16.7%)	2 (25.0%)	1 (16.7%)	1 (20.0%)	**5 (20.0%)**
Chemotherapy of radiation therapy	1 (16.7%)	1 (12.5%)	0	0	**2 (8.0%)**
Pre‐op or pre‐procedure for invasive procedures/surgeries	1 (16.7%)	0	0	0	**1 (4.0%)**
Patient being administered Coumadin	0	1 (12.5%)	0	0	**1 (4.0%)**
Hospice care	0	1 (12.5%)	0	0	**1 (4.0%)**
Person without capacity to make an informed decision	0	1 (12.5%)	0	0	**1 (4.0%)**
Physician discretion	0	0	1 (16.7%)	0	**1 (4.0%)**
Recent pneumonia hospitalization	0	0	2 (33.3%)	0	**2 (8.0%)**
