# Supplementary material for: Assessing the prior event rate ratio method via probabilistic bias analysis on a Bayesian network
Source: Stat Med. 2019 Dec 1;39(5):639–59. doi: 10.1002/sim.8435 (PMC7027899; doi:10.1002/sim.8435)
Supplement: Supplementary file 1 — SIM_8435‐Suppl‐0001.zip [file SIM-39-639-s001.zip › Readme.docx]

## Using Bayesian network probabilistic bias analysis R code

### Test run: Conduct PBA, produce a version of Figure 8 in the paper, and print out some statistics

1. Install R packages “doSNOW”, “foreach”, “ggplot2”, “gridExtra”, “cowplot”, “ggpubr”, “DescTools”, “canprot” and “parallel” if they are not currently installed.
2. Make sure the R scripts **analytic_DAG_v7.0.R, PBA_tools.R, Fig8_inputs.R** and **run_PBA_and_plot.R** are in R’s current working directory.
3. Determine your computer’s number of cores:

> library(parallel)

> detectCores()

1. Modify Line 31 of **analytic_DAG_v7.0.R** to the number of cores reported in Step 3. For example, if detectCores() returned “4”, then Line 31 should be

cl <- makeCluster (10)

1. Modify Line 1 of **run_PBA_and_plot.R** to set variable Nreps to the desired number of Monte Carlo realizations. Set to 1000 by default (which should run quite fast). For Figure 8 of the manuscript, Nreps was set to 50000.
2. Source the R script that runs everything:

> source('./run_PBA_and_plot.R’)

The following should happen:

- a frame called “frame_fig8” is produced in the R environment
- an PNG image called “Fig8.png” is created in the R working directory.
- some statistics describing the PBA results vs. the Young-Xu et al. (2018) PERR results are printed to the R console
